# Improved crop yield and reduced nitrate nitrogen leaching with straw return in a rice-wheat rotation of Ningxia irrigation district

**DOI:** 10.1038/s41598-018-27776-5

**Published:** 2018-06-21

**Authors:** Shiqi Yang, Yongsheng Wang, Ruliang Liu, Lei Xing, Zhengli Yang

**Affiliations:** 1 Institute of Environment and Sustainable Development in Agriculture, Chinese Academy of Agricultural Sciences, Beijing, 100081 China; 2 Key Laboratory of Agro-Environment and Climate Change, Ministry of Agriculture, Beijing, 100081 China; 3 Key Laboratory of Regional Sustainable Development Modeling, Institute of Geographic Sciences and Natural Resources Research, Chinese Academy of Sciences, Beijing, 100101 China; 4 Ningxia Academy of Agriculture and Forestry Science, Yinchuan, 750002 China; 5 Present Address: No. 11, Datun road, Chaoyang district, Beijing, 100101 China

## Abstract

Field experiments were conducted in rice-wheat rotation under conventional management to determine the effects of straw return ((half straw return, HS) and (total straw return, TS)) on crop yield, N uptake, soil properties and soil NO_3_^−^–N leaching. We found that straw return significantly increased crop yield and N uptake. TS significantly increased soil SOM at depths of 20 cm and 30 cm. Straw return had significantly increased soil NO_3_^−^–N leaching at a depth of 10 cm, whereas significantly decreased soil NO_3_^−^–N leaching at depths of 30 cm and 90 cm in the rice season. In wheat season, HS and TS performed better than conventional fertilization management without straw return in reducing soil NO_3_^−^–N leaching at depth of 90 cm. Soil NO_3_^−^–N leaching was significantly decreased through enhancing total N uptake, improving soil aggregation and decreasing soil NO_3_^−^–N concentration. Our results indicated that total straw return has the potential to increase crop yield, improve soil aggregation and decrease soil NO_3_^−^–N concentration, thus increasing total N uptake and reducing soil NO_3_^−^–N leaching in the rice-wheat rotation system of Ningxia Yellow river irrigation district. In the future, the long-term observation of crop yield and nitrate nitrogen leaching are necessary to identify the environmentally friendly straw return practices for rice-wheat rotation.

## Introduction

Synthetic nitrogen (N) fertilizer has enabled the doubling of world food production in the past four decades^1^. However, excessive fertilizer N inputs with decreasing N use efficiency have resulted in environmental pollution problems, such as leaching of nitrate and emission of nitrous oxide and ammonia^2^. China’s field experiments have shown low nitrogen use efficacy of 26–28% in 2001–2005 for major cereal crops^3^, relative to 52% in America and 68% in Europe^4^. Nitrogen leaching in China (13.7–347 kg N ha^−1^) was significantly higher than that of Europe and America (4–107 kg N ha^−1^)^5^. Nitrate nitrogen (NO_3_^−^–N) leaching has been of major concern in China recent decades due to its harmful effect on groundwater and human health^6^. Therefore, knowing how to effectively control soil NO_3_^−^–N leaching has become an important issue for the development of sustainable agriculture.

Soil NO_3_^−^–N leaching is greatly influenced by edaphic and climatic factors and agricultural management practices^7^. Straw are the primary source of N for the microbial biomass and for plants^8^. China produces the most crop residue in the world, approximately 8 × 10^10^ kg yr^−1^. However, 32.3% of crop residues are directly used as fuels, 27.1% are used as feed, 16.8% are discarded or burnt in open fields and only 14.1% are returned to the soil^9^. Straw return may improve soil structure and retain water^10^. Enhanced N mineralization and N use efficiency and reduce N leaching were reported after amending soil with straw in the previous studies^7,11,12^. However, greater amounts of straw return or deeper burial depth resulted in more water percolation and N leaching^13^. Appropriate straw return methods might be effective mitigation strategies for controlling N leaching in agricultural soils.

Ningxia irrigation region is one of the oldest and largest irrigation areas in northwest China, and sustains over 60% of the Ningxia population. From 1978 to 2009, total annual grain production and chemical fertilizer consumption increased 2.84 times and 4.24 times, respectively^14^. Currently, the conventional application rate of synthetic N fertilizer is 300 kg ha^−1^ yr^−1^ for paddy due to flooding irrigation. Higher N fertilizer rate will inevitably promote the N leaching into the water bodies, and lead to the non-point source pollution to the Yellow River^15,16^. Annual N loss in Qingtongxia area of Yellow River was 41.1 thousand tons, which is 1.52 times higher than that from the interzone point source pollution^17^. Both total N and ammonium N contents were increasing significantly with the increase of fertilizer application rates, especially since 1990s in Ningxia segment of the Yellow River^18,19^. It is essential to explore the agricultural management measures for reducing N leaching from farmland in Ningxia irrigation region.

Rice-wheat rotation is the dominant farming system in the Ningxia Yellow river irrigation region of China. Unfortunately, high N fertilizer application resulted in the higher N surplus in soil due to the reduced rice N uptake derived from soil in Ningxia Yellow river irrigation^16^. Many methods are studied to reduce N leaching losses, while not impairing the crop yields to ensure food security in Ningxia, including reducing and postponing N application^20^, side-dressing^15^, biochar and manure amendment^21,22^. In 2008, the total straw amounts of wheat, rice and maize from Ningxia province was estimated to be 70.48 × 10^7^ kg, 73.02 × 10^7^ kg and 164.94 × 10^7^ kg, respectively^23^. Previous study showed higher overall straw utilization rate of 68.9% in Ningxia provinces, compared with 35.0% of national level^24^. However, only 2.9% of total straw was returned to agricultural soil and used as fertilizer^23^. Return of straw with N fertilizer will has a widespread prospect in agricultural field management to increase productivity and reduce soil NO_3_^−^–N leaching in Ningxia area^25,26^. However, the effects of straw return on soil NO_3_^−^–N leaching of rice-wheat rotation system were not unclearly. We hypothesized that straw return will increase crop yields and reduce soil NO_3_^−^–N leaching in rich-wheat rotation, and this response will promoted with increasing amounts of straw return. In this study, our objectives were (1) to examine the effects of straw return on rice-wheat rotation system crop yields as well as soil NO_3_^−^–N leaching; and (2) to identify the environmental friendly straw return management practices for rice-wheat system in Ningxia region.

## Materials and Methods

### Study site

This study was conducted at Lingwu Farm (38°07′14″N, 106°17′43″E) in Yinchuan City, China. The temperate continental monsoon climate dominates the region, with a mean temperature of 8.9 °C and a mean annual precipitation of 192.9 mm. The soil is classified as anthropogenic alluvial soil, with a soil texture of 18.25% clay, 53.76% silt, and 27.99% sand. The top soil (0–30 cm) organic matter is 10.58 g kg^−1^, the total N is 0.98 g kg^−1^, and the soil bulk density is 1.39 g cm^−3^.

### Experimental design and agricultural management

The straw return experiment is a randomized block design with three treatments: CM (Conventional fertilization management without straw return), HS (Conventional fertilization management with half straw return, about 0.40 kg m^−2^ rice straw or 0.20 kg m^−2^ wheat straw), TS (Conventional fertilization management with total straw return, about 0.80 kg m^−2^ rice straw or 0.40 kg m^−2^ wheat straw). Each treatment was performed in triplicate. A total of 9 plots (10 m × 30 m) were established.

Urea was applied at 300 kg N ha^−1^ and 225 kg N ha^−1^ for rice and wheat, respectively, of which 10% was applied before winter irrigation, 50% was applied as a base fertilizer, 30% was applied at the tillering stage, and the remaining 10% was applied at the elongation stage. Double superphosphate and KCL were also applied as basal fertilizers at rates of 105 kg P_2_O_5_ ha^−1^ and 60 kg K_2_O ha^−1^, respectively. Rice and wheat straw was cut to 6–8 cm by harvester during the harvest period. Each plot was irrigated with an equal amount of water. Total frequency of water irrigation was 16 times and 4 times, with total amount of 14500 m^3^ ha^−1^ yr^−1^ and 4350 m^3^ ha^−1^ yr^−1^ for rice and wheat season, respectively (Table 1). Straw and fertilizers were broadcast on the soil surface and incorporated into the soil by plowing to a depth of approximately 10 cm before winter irrigation. Other crop management was consistent across plots in each crop season. The experiment was carried out over 2 years beginning in 2010 and ending in 2011.

**Table 1:** Frequency and total volume of water irrigation during rice-wheat rotation period.

Growth stage	Tillering (time)	Elongation (time)	Booting (time)	Filling (time)	Fallow (time)	Total volume (m^3^ ha^−1^)
Rice	8	3	3	1	1	14500
Wheat	1	1	1	1	0	4350

### Soil NO_3_^−^–N losses measurement

Soil NO_3_^−^–N leaching losses was measured by the method of exchange resin core^26^. Four tubes (stainless steel, 43 cm^2^) were installed at the desired depth (10, 20, 30 and 100 cm) below the soil surface for each treatment condition. About 2 cm soil was removal from the tube bottom after getting the intact soil core by the above tube. About 15 g of anion ion exchange resin (SIGMA, USA) was packed into nylon bag (41 cm^2^) and put at the tube bottom. Primary soil was used to fill the tube bottom and prevent the resin bag being dropped^26^. Intact soil core was inserted and cultivated *in situ* without crop in it. Soil leachate NO_3_^−^–N was absorbed by anion ion exchange resin during migration in the soil profile and was desorbed using 2 M KCL solution. The NO_3_^−^–N leaching losses were calculated by multiplying the N concentration by the desorbed solution volume.

### Rice yield and N uptake

At crop maturity, crop aboveground biomass was estimated by manually harvesting three 0.5 m^2^ areas. Straw and grain were oven-dried to a constant weight at 70 °C^16^, weighed, finely ground, sieved, and analyzed for total N using the Kjeldahl method^27^. The uptake of N in straw and grain was calculated by multiplying yield of straw and grain, respectively, with N concentrations in straw and grain. Total N uptake was calculated from the sum of the N mass in the straw and grain harvested from each plot.

### Soil sampling and analysis

Soil samples were taken at 10 cm intervals to a depth of 30 cm using an auger. Three soils from each plot were collected and pooled into one composite sample. Soil samples were collected seven times and three times in the critical growth stage of previous rice season and later wheat season, respectively. Soils were sieved to 2-mm mesh size in the field and were then transported to the lab in a biological refrigerator. Soil samples were stored at −20 °C before analysis. Soil NO_3_^−^–N concentration was determined using a continuous-flow auto analyzer (Seal AA3, Germany). The total N (TN) contents in the bulk soil were determined by dry combustion using the Kjeldahl method^27^. Soil organic carbon concentration was determined using K_2_Cr_2_O_7_ oxidation method. Active soil organic carbon was determined using the following method^28^. Soil was treated with 25 ml 333 mM KMnO_4_ and shaken for 6 h and centrifuged for 5 min at 33.3 rps. The absorbance of the supernatant and standards were read at 565 nm. The change in the concentration of KMnO_4_ was used to estimate the amount of carbon oxidized, assuming that 1 mM KMnO_4_ is consumed in the oxidation of 9 mg of carbon. Soil organic matter (SOM) and active soil organic matter (ASOM) was all calculated using the efficiency factor of 1.724^27^. In wheat season, soil bulk density was measured using a 100 cm^3^ cylinder. Soil porosity was measured by the difference between saturated and dry soil weight of known volumes^29^.

### Statistical analyses

Repeated measures of analysis of variance (ANOVA) with the least significant difference (LSD) test were applied to examine the differences in soil NO_3_^−^–N leaching among the different treatments. Straw return was set as a between-subjects factor, and the measurement period was selected as a within-subjects variable. We performed one-way ANOVA with an LSD test to evaluate the effects of straw return on the soil properties, crop yield and N uptake. All statistical analyses were conducted using the SPSS software (version 16.0), and differences were considered significant at *P* < 0.05, unless otherwise stated. All figures were drawn using SigmaPlot software (version 10.0).

## Results

### Crop yield and N uptake

In the CM, the rice yield and wheat yield was 6358 kg ha^−1^ and 3749 kg ha^−1^, respectively. Straw return significantly increased the crop yield compared with the CM treatment for the HS and TS treatments (Table 2). Grain and straw N uptake increased with increasing straw return amount. The relative increase induced by straw return on total rice N uptake was 10.43% and 22.02% for the HS and TS treatments, respectively. In addition, total wheat N uptake was increased by 10.69% and 12.18% in HS and TS, respectively, compared with CM (Table 2).

**Table 2:** Yield and N uptake under different experimental treatments (n = 3).

Treatment		Crop yield (kg ha^−1^)	Grain N uptake (kg ha^−1^)	Straw N uptake (kg ha^−1^)	Total N uptake (kg ha^−1^)
Rice	CM	6357 ± 136c	66.34 ± 1.72c	55.10 ± 1.02c	121.44 ± 2.74c
	HS	6952 ± 93b	72.77 ± 1.39b	61.34 ± 1.43b	134.11 ± 1.81b
	TS	7447 ± 143a	76.20 ± 1.24a	71.98 ± 2.32a	148.18 ± 2.87a
Wheat	CM	3749 ± 115b	79.72 ± 2.34c	35.29 ± 0.13b	115.01 ± 2.42b
	HS	4154 ± 84a	88.34 ± 1.80a	38.97 ± 0.70a	127.31 ± 2.46a
	TS	4198.73 ± 86a	89.44 ± 1.99a	39.57 ± 0.34a	129.01 ± 2.33a

### Soil properties

Straw return significantly decreased soil bulk density at the 0–10 cm depth only (Table 3). Soil porosity in TS treatment was significantly higher than that of CM treatment at the 0–10 cm depth. However, the effects of straw return on soil bulk density and soil porosity were not significantly between the 10–20 cm and 20–30 cm depth (Table 3).

**Table 3:** Soil bulk density and porosity under different experimental treatments (n = 3).

Treatment	Soil bulk density (g cm^−3^)			Soil porosity		
	0–10 cm	10–20 cm	20–30 cm	0–10 cm	10–20 cm	20–30 cm
CM	1.53 ± 0.03a	1.51 ± 0.05a	1.59 ± 0.04a	0.42 ± 0.02 b	0.40 ± 0.04a	0.41 ± 0.02a
HS	1.48 ± 0.02 b	1.54 ± 0.02a	1.60 ± 0.01a	0.44 ± 0.01ab	0.41 ± 0.05a	0.38 ± 0.06a
TS	1.49 ± 0.02 b	1.57 ± 0.03a	1.65 ± 0.04a	0.45 ± 0.02a	0.39 ± 0.03a	0.36 ± 0.03a

**Figure 1:**
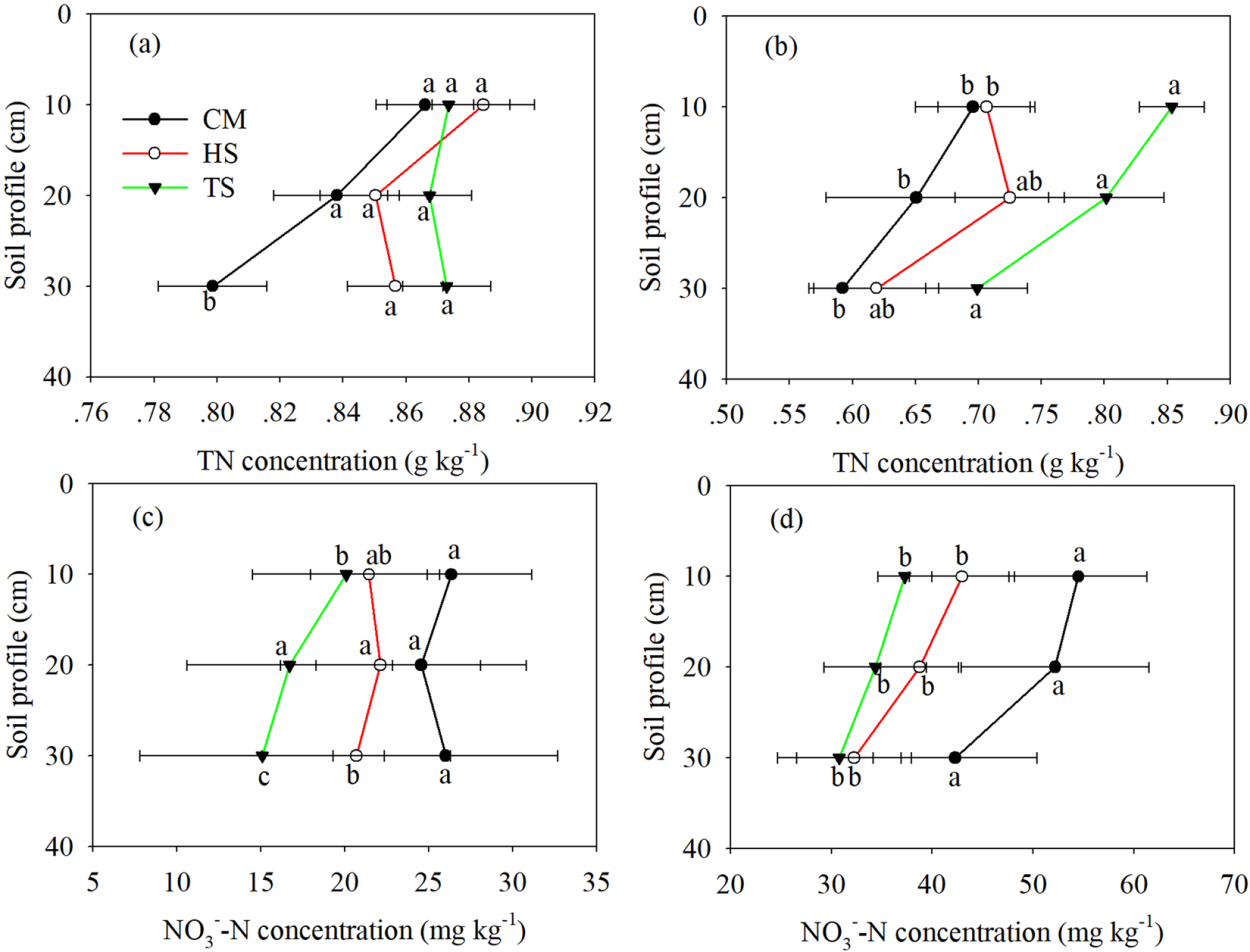
Arithmetic means of soil TN and soil NO_3_^−^–N concentrations under the experimental treatments in rice (**a,c**) and wheat (**b,d**) season. Data are shown as means with standard errors (n = 3). Different letters below the columns mean significant difference among the treatments.

In rice season, relative to the CM, HS and TS increased rice soil TN concentration by 7.51% and 8.76% at a depth of 30 cm, respectively (Fig. 1a). The lowest soil NO_3_^−^–N concentration occurred in the TS treatment at a depth of 10 cm (Fig. 1c). Furthermore, HS and TS treatments significantly decreased soil NO_3_^−^–N concentration at a depth of 30 cm (Fig. 1c). In wheat season, straw return increased TN concentration, while significantly decreased wheat soil NO_3_^−^–N concentration at various depths (Fig. 1b, Fig. 1d).

**Figure 2:**
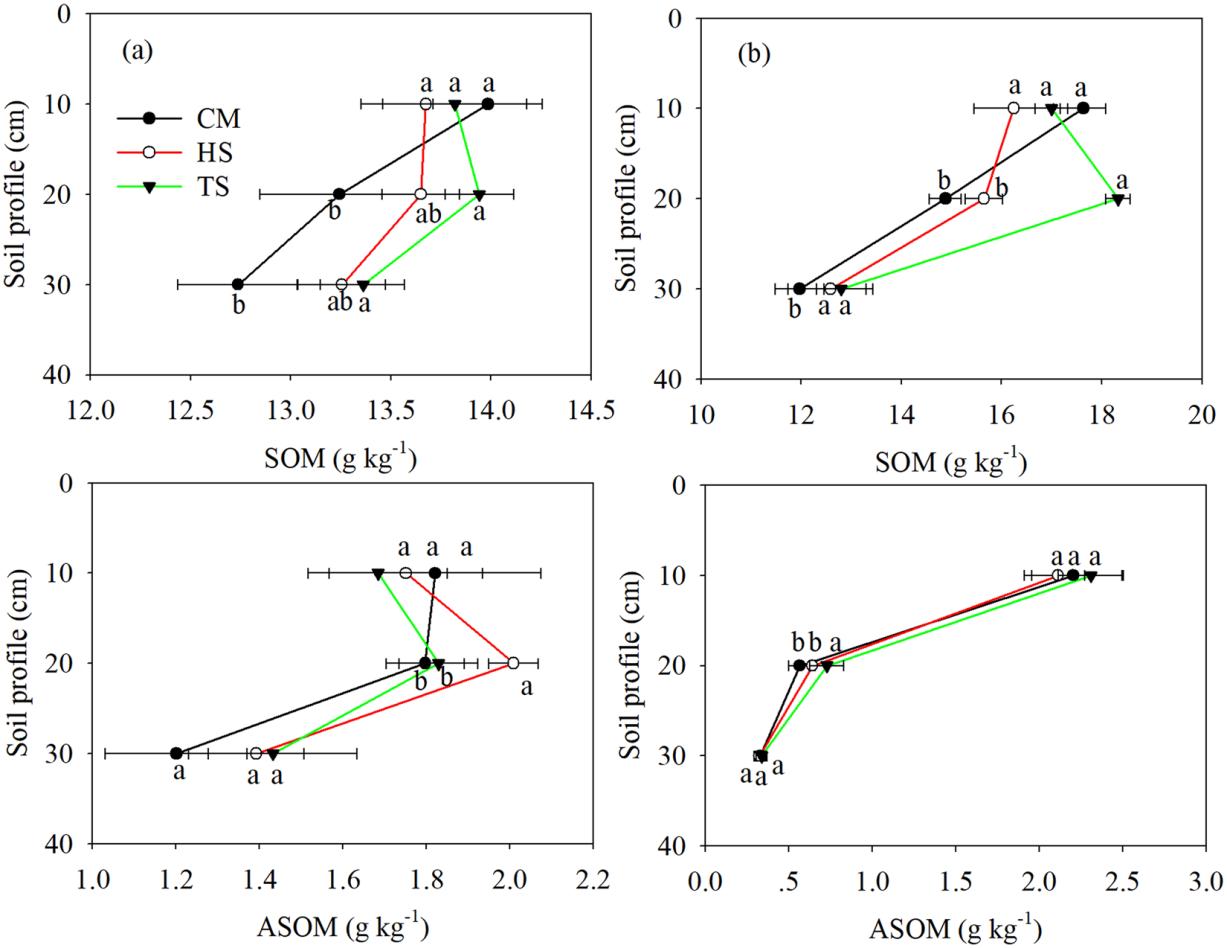
Arithmetic means of SOM and ASOM concentrations under the experimental treatments in rice (**a,c**) and wheat (**b,d**) season. Data are shown as means with standard errors (n = 3). Different letters below the columns mean significant difference among the treatments.

Except for the depth of 10 cm, TS significantly increased SOM in the rice season (Fig. 2a). In the wheat season, SOM of TS treatment significantly higher than that of CM and HS treatments at a depth of 20 cm. Straw return significantly increased wheat SOM at a depth of 30 cm (Fig. 2b). The significantly difference of ASOM was only found at a depth of 20 cm with the highest value occurred in the HS and TS treatments in rice and wheat season, respectively (Fig. 2c-d).

### Soil NO_3_^−^–N leaching

**Figure 3:**
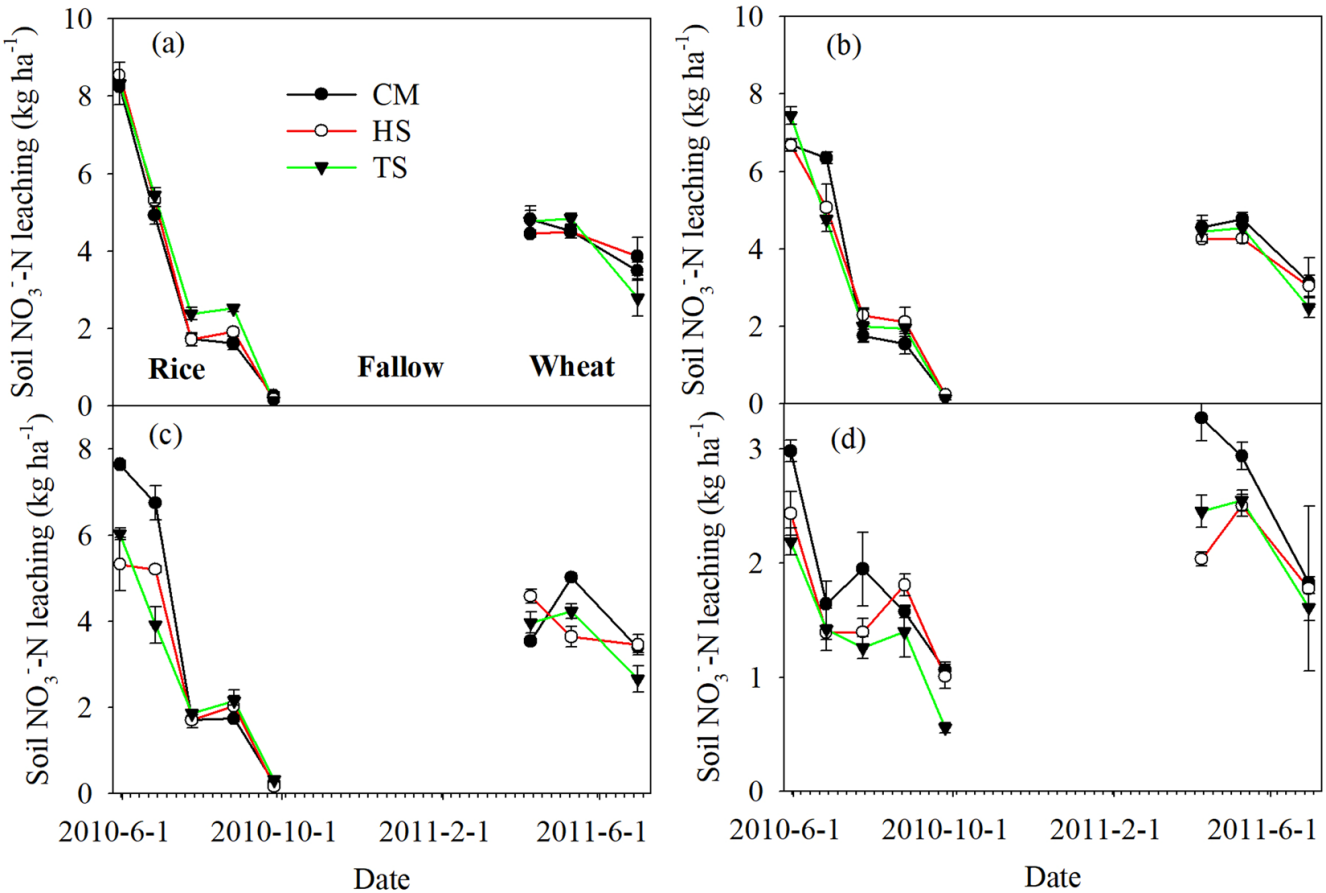
Variation of soil NO_3_^−^–N leaching at various soil depths under the experimental treatments. Data are shown as means with standard errors (n = 3). Different letters below the columns mean significant difference among the treatments. Date format is Year-Month-Date.

**Figure 4:**
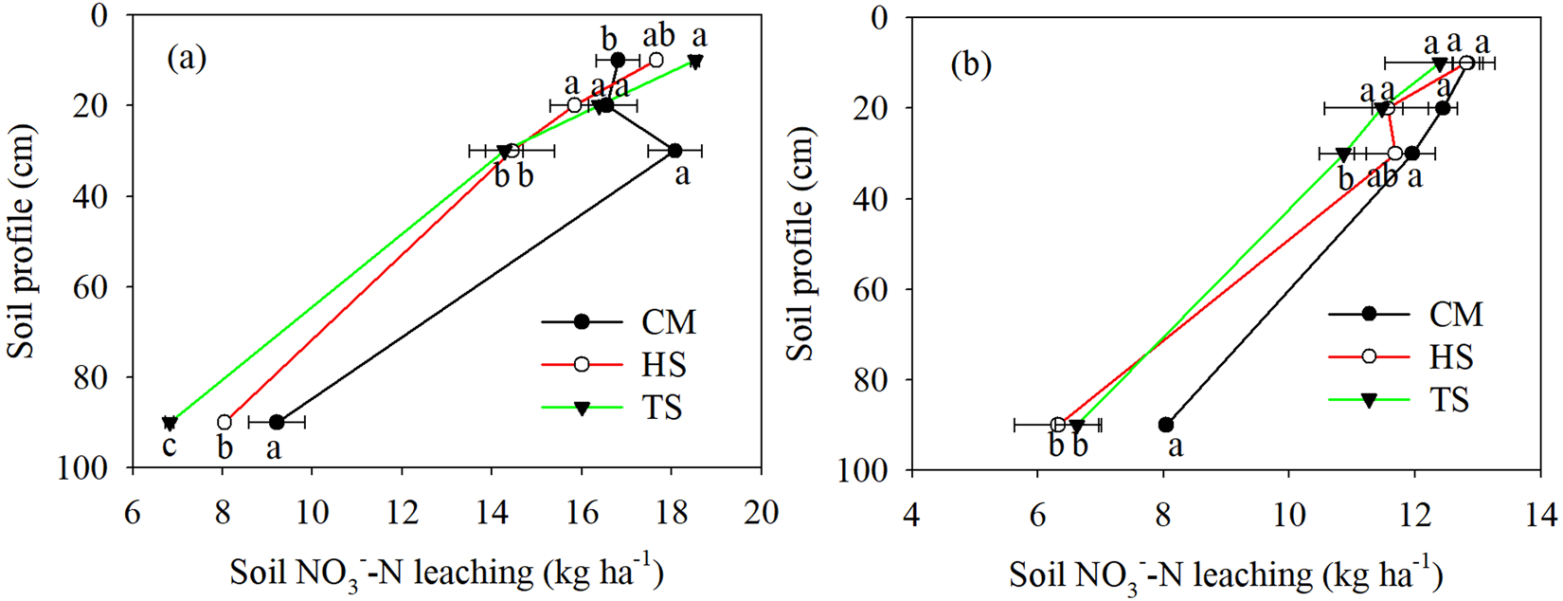
Arithmetic means of soil NO_3_^−^–N leaching under the experimental treatments in rice (**a**) and wheat (**b**) season. Data are shown as means with standard errors (n = 3). Different letters below the columns mean significant difference among the treatments.

Soil NO_3_^−^–N leaching was significantly decreased with crop growth at various soil depths (Fig. 3, Table 4). The new soil NO_3_^−^–N leaching peak was occurred at depth of 90 cm in rice season (Fig. 3d). The averaged soil NO_3_^−^–N leaching decreased with the soil depth (Fig. 4).

**Table 4:** Results of repeated measures ANOVA on the effects on straw return, date, and their interaction on soil NO_3_^−^–N leaching (n = 3).

Source of variation		Depth (cm)			
		10	20	30	90
Rice	Date	<0.001	<0.001	<0.001	<0.001
	Treatment	0.317	0.631	0.013	0.011
	Date × Treatment	0.014	0.007	<0.001	0.060
Wheat	Date	<0.001	<0.001	<0.001	0.002
	Treatment	0.811	0.447	0.250	0.049
	Date × Treatment	0.117	0.681	<0.001	0.338

Straw return had significantly increased soil NO_3_^−^–N leaching at a depth of 10 cm, whereas significantly decreased soil NO_3_^−^–N leaching at depths of 30 cm and 90 cm in the rice season (Fig. 4a). Furthermore, significant interactions were found between the observation date and straw return treatment, except for the depth of 90 cm (Table 4, *P* < 0.001). In wheat season, HS and TS showed significantly decreased soil NO_3_^−^–N leaching at depth of 90 cm, compared with CM (Fig. 4b). The significant interaction for soil NO_3_^−^–N leaching was only found at depth of 30 cm between observation date and straw return treatment (Table 4, *P* < 0.001).

## Discussions

### Effects of straw return on crop yield

The effects of straw return on crop yield is still under debate since field results across various pedo-climatic environments are inconclusive, partly due to the numerous and complex factors that affect the straw-derived N cycle under field conditions^30^. Crop yield benefits from straw return are seen in N-restricted or over-fertilization in the North China Plain^31^. In our study, HS and TS treatments significantly increased the rice yield by 9.35% and 17.15%, respectively, while the increases in wheat yield elicited by HS and TS return were 10.80% and 12.00%, respectively (Table 2). Enhanced crop yields to straw return could be related to the following aspects. In Ningxia irrigation region, over use of N fertilizer in the rice-wheat rotation system has resulted in soil NO_3_^−^–N leaching out of root zone^16,20^. The N immobilized by straw would be released across crop growing season, thus improving N uptake (Table 2) and crop yield^32^. Yang *et al*. also suggested that ditch-buried straw return has the potential to increase crop N uptake and crop yield in the rice-wheat system through increased N retention in the soil. In addition, straw benefit the improvement of soil properties, such as soil bulk density, soil porosity and SOM (Table 3), and consequently promoting the improvement of crop yield^13,32^. Therefore, our preliminary results revealed that crop yield improvement is attributed to both increased soil TN and additional nutrients supplement after straw return. However, the meta-analysis and long-term results revealed that incorporation of crop straw produced no significant trend in improving crop yield. Therefore, the effects of straw return on rice-wheat yields were needed further study^34,35^.

### Effects of straw return on soil NO_3_^−^–N leaching

Organic amendments are often shown to increase soil nitrogen retention and reduce N leaching^21,36^. In our study, the significantly reductions in soil NO_3_^−^–N leaching were observed at depths of 30 cm and 90 cm in rice season and at depth of 90 cm in wheat season (Fig. 4). This result would be expected and is in accord with several previous studies^11,13^. First, the moderate increase in the N use efficiency may be associated with higher reduction rate of N leaching. The promotion of crop N uptake is critical to reduce N pollution in agro ecosystems due to minimizing surplus soil N^35^. In the study area, evidence for the decreased soil NO_3_^−^–N leaching was provided by the increased total N uptake after straw return (Table 2). Second, large quantity of straw return may strongly physically absorb N and alter N spatial distribution in the soil profile. Wheat straw carries negative charges and shows good adsorptive capability for urea-N^37^. Otherwise, straw fixed part of the NO_3_^−^–N and released organic acids during its decomposition and inhibited the transformation of NH_4_^+^–N to NO_3_^−^–N^38^. In our experiment, straw return tended to increase TN concentration while decrease soil NO_3_^−^–N concentration in the upper 30 cm soil (Fig. 1). Thus more available N in the upper part of soil can be preferentially utilized by crops, and few soil NO_3_^−^–N leaching below the rooting zone. Third, the increased availability of carbon source following straw return, may stimulate the dissimilatory NO_3_^−^–N reduction to NH_4_^+^–N (DNRA) and therefore promoting N retention in soil and reducing soil NO_3_^−^–N leaching and runoff^39^. Increased SOM and higher ASOM (Fig. 2) in our straw return treatments may have promote microbial growth and serve to immobilize N^40^. Meanwhile, the large available carbon could prime nitrifies and denitrifies, which could contribute to N loss as gaseous emissions. Moreover, the high C/N ratio of straw incorporated into the soil can transform mineral N to organic N by immobilization^41^. Finally, the extension of fungal hyphal by straw return could improve soil aggregation, thus enhancing water infiltration^42^. The increased soil water holding capacity due to the reduced soil bulk density and increased soil porosity (Table 2) may have also reduced soil NO_3_^−^–N leaching. Overall, straw return reduced the soil NO_3_^−^–N leaching via promotion total N uptake, reducing availability of soil NO_3_^−^–N concentration and SOM-induced N immobilization. It is noted that the concentration of N fractions in irrigation water will influence soil properties and chemistry as well as soil NO_3_^−^–N leaching. In our study, this effect can be eliminated due to the same water source in Yellow River, equal irrigation time and amount for each plot (Table 1). In addition, reduced soil NO_3_^−^–N concentration suggests that the concentrations of soil dissolved organic N or NH_4_^+^-N may increase in the upper 30 cm. Therefore, effective managements such as minimized soil disturbance, lower winter irrigation in the fallow period are beneficial to keep soil N pools from loss via deep leaching or gases emission.

## Conclusions

The effects of straw return on crop yield, N uptake, soil properties and soil NO_3_^−^–N leaching were investigated in rice-wheat rotation system in Ningxia Yellow river district. Straw return significantly increased N uptake, soil porosity, TN concentration, SOM and ASOM contents, but it significantly decreased soil bulk density and soil NO_3_^−^–N concentration. Straw return significantly increased crop yields and N uptake. Soil NO_3_^−^–N leaching was significantly decreased through enhancing total N uptake, improving soil aggregation and decreasing soil NO_3_^−^–N concentration. In summary, our study has shown that total straw return about showed a good promotion of crop yield and good reduction in soil NO_3_^−^–N leaching in the rice–wheat rotation system in Ningxia Yellow river irrigation district. However, this is only two years results with one rice-wheat rotation. The responses of crop yield and soil NO_3_^−^–N leaching to straw return were also influenced by the interannual variability in precipitation and temperature. Long-term study should be enhanced to identify the environmentally friendly straw return practices for rice-wheat rotation.
